# The Value of Serum MicroRNA Expression Signature in Predicting Refractoriness to Bortezomib-Based Therapy in Multiple Myeloma Patients

**DOI:** 10.3390/cancers12092569

**Published:** 2020-09-09

**Authors:** Paweł Robak, Izabela Dróżdż, Dariusz Jarych, Damian Mikulski, Edyta Węgłowska, Monika Siemieniuk-Ryś, Małgorzata Misiewicz, Konrad Stawiski, Wojciech Fendler, Janusz Szemraj, Piotr Smolewski, Tadeusz Robak

**Affiliations:** 1 Department of Experimental Hematology, Medical University of Lodz, 93-510 Lodz, Poland; pawel.robak@umed.lodz.pl (P.R.); piotr.smolewski@umed.lodz.pl (P.S.); 2 Department of Clinical Genetics, Medical University of Lodz, 92-213 Lodz, Poland; izabela.drozdz@umed.lodz.pl; 3 Laboratory of Personalized Medicine, Bionanopark, Lodz, 93-465 Lodz, Poland; d.jarych@bionanopark.pl (D.J.); e.weglowska@bionanopark.pl (E.W.); 4 Department of Biostatistics and Translational Medicine, Medical University of Lodz, 92-215 Lodz, Poland; damian.mikulski@stud.umed.lodz.pl (D.M.); konrad.stawiski@stud.umed.lodz.pl (K.S.); wojciech_fendler@dfci.harvard.edu (W.F.); 5 Department of Hematology, Medical University of Lodz, 93-510 Lodz, Poland; monika.siemieniuk-rys@umed.lodz.pl (M.S.-R.); malgorzata.misiewicz@umed.lodz.pl (M.M.); 6 Department of Medical Biochemistry, Medical University of Lodz, 92-215 Lodz, Poland; janusz.szemraj@umed.lodz.pl

**Keywords:** bortezomib, efficacy, multiple myeloma, resistance, sensitivity, refractory, microRNA, miR-215-5p, miR-181a-5p, miR-376c-3p

## Abstract

**Simple Summary:**

The proteasome inhibitor bortezomib is currently commonly used for the treatment of multiple myeloma (MM). MicroRNAs (miRNAs) are small non-coding RNAs that play a crucial role in messenger RNA silencing and post-transcriptional regulation of gene expression. In MM, the expression of several miRNAs is markedly dysregulated suggesting their role in MM pathogenesis and drug resistance. The aim of our study was to assess miRNA expression patterns in the serum of MM patients treated with bortezomib. We have identified 21 serum miRNAs differentially expressed in patients refractory to bortezomib-based chemotherapy. A miRNAs-based prediction model was developed to assess the probability of refractoriness to bortezomib. Our findings, indicating the differential expression of miRNAs between bortezomib-refractory and bortezomib-sensitive patients, suggest that these circulating miRNAs may play an important role in personalized treatment of MM patients.

**Abstract:**

Bortezomib is the first-in-class proteasome inhibitor, commonly used in the treatment of multiple myeloma (MM). The mechanisms underlying acquired bortezomib resistance in MM are poorly understood. Several cell-free miRNAs have been found to be aberrantly regulated in MM patients. The aim of this pilot study was to identify a blood-based miRNA signature that predicts bortezomib-based therapy efficacy in MM patients. Thirty MM patients treated with bortezomib-based regimens were studied, including 19 with refractory disease and 11 who were bortezomib sensitive. Serum miRNA expression patterns were identified with miRCURY LNA miRNA miRNome PCR Panels I+II (Exiqon/Qiagen). Univariate analysis found a total of 21 miRNAs to be differentially expressed in patients with MM according to bortezomib sensitivity. Multivariate logistic regression was created and allowed us to discriminate refractory from sensitive patients with a very high AUC of 0.95 (95%CI: 0.84–1.00); sensitivity, specificity and accuracy were estimated as 0.95, 0.91, and 0.93. The model used expression of 3 miRNAs: miR-215-5p, miR-181a-5p and miR-376c-3p. This study is the first to demonstrate that serum expression of several miRNAs differs between patients who are bortezomib refractory and those who are sensitive which may prove useful in studies aimed at overcoming drug resistance in MM treatment.

## 1. Introduction

Multiple myeloma (MM) is a hematological malignancy characterized by the clonal proliferation of plasma cells in the bone marrow (BM) microenvironment [1,2]. It is the second most prevalent hematological malignancy, accounting for 1.3% of all malignancies and 15% of hematological neoplasms, with an annual incidence of 4.5–6 cases per 100,000 people [3].

Multiple myeloma often results in bone lesions, hypercalcemia, infections, anemia and production of monoclonal immunoglobulin. The interaction of MM cells with host factors, and the BM microenvironment plays a key role in the molecular evolution of the disease and the generation of treatment-resistant cells; it also influences disease progression with the development of relapsed or refractory disease [4,5]. The treatment of MM has changed in recent years, with the introduction of new drugs, most notably, proteasome inhibitors such as bortezomib, carfilzomib and ixazomib, immunomodulatory drugs (lenalidomide, pomalidomide), a histone deacetylase inhibitors (panobinostat) and monoclonal antibodies (elotuzumab and daratumumab) [6,7]. Bortezomib inhibits the proteasomal degradation of several regulatory ubiquitinated proteins and exerts substantial anti-myeloma activity in previously untreated and relapsed/refractory MM patients. The drug can be used as a single agent but is more frequently administered in combination with other anti-myeloma drugs [8]. However, the therapeutic resistance of these agents, which emerges soon after the onset of therapy in some cases, limits the efficacy of the treatment in most patients, even if they initially responded to therapy [9].

MicroRNAs (miRNAs) are small, non-coding RNAs only 19 to 25 bases in length that are secreted into the circulation and influence the regulation of gene expression in physiological or pathological conditions [10,11,12]. Many studies have shown that miRNAs influence on several biological processes associated with cancers, such as cancer proliferation, apoptosis, migration and invasion [13]. MiRNAs can regulate the expression of tumor suppressor genes or oncogenes and play a functional role in cancer progression. The profile of cell-free miRNAs or circulating miRNAs could be used as a signature of disease status, including MM. Some studies indicate lowered expression of several miRNAs, including miR-125b, miR-133a, miR-1, miR-124a, miR-15, and miR-16 in cell samples from patients with MM than in normal individuals [14,15]. In contrast, higher expression of miR-21 has been observed in MM plasma cells compared to plasma cells from individuals without MM [16]. Gupta al. found miR-203 to demonstrate high sensitivity (83%) and specificity (83%) in the diagnosis of MM [17].

In patients with MM, miRNAs seem to be involved in drug resistance [15,18,19,20,21]. For example, miR-21, miR-221/222, miR-125a and miR-125b, and miR-451 are upregulated in MM cells resistant to antimyeloma drugs [21,22,23,24]. MiRNAs play an important role in regulating drug resistance by directly targeting oncogenes and tumor suppressor genes in neoplastic diseases [23]. However, very little data exists about the types of circulating miRNA in MM patients who are sensitive, or refractory, to bortezomib. Identifying the miRNAs which mediate resistance of MM cells to targeted therapy may improve proper treatment selection, overall response (OR) and survival. The present study analyzes serum miRNA expression patterns in the serum of MM patients subsequently treated with bortezomib-based regimens.

## 2. Results

In Table 1 we present the demographic, clinical and laboratory characteristics of the 30 MM patients enrolled for the study. A total number of 18 men and 12 women were studied and the mean age of study population was 64.0 ± 9.9 years (range 39–81). The *predominant paraprotein* detected was IgG type (60.0%) followed by light chain myeloma (23.3%). Most of the patients received a bortezomib, cyclophosphamide and dexamethasone (VCD) regimen; however, two patients received VMP (bortezomib, melphalan and prednisone), and one received VTD (bortezomib, thalidomide, dexamethasone). Nineteen patients were refractory and 11 sensitive to bortezomib-based therapies. Overall, no statistically significant differences were observed between the refractory and sensitive group regarding the studied clinical variables (Table 1).

**Figure 1:**
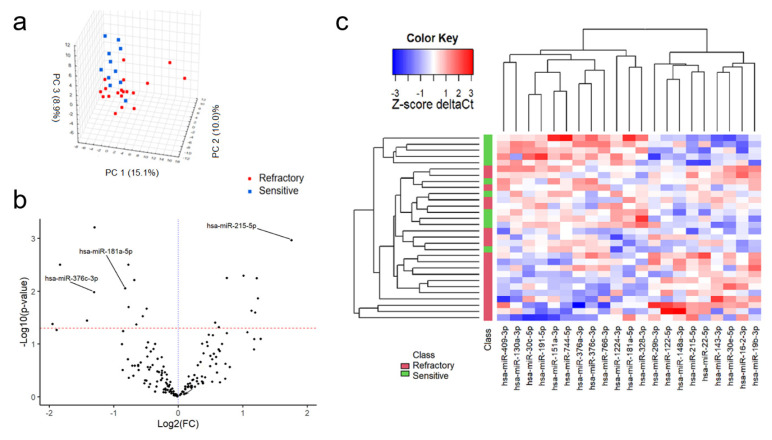
Comparision of miRNA expression between MM patients sensitive and refractory to bortezomib-based treatment. 3D plot of principal comptonent analysis (PCA) score performed on all 155 miRNAs (**a**). Volcano plot of filtered miRNAs. miRNAs included in the logistic regression model are indicated by their labels (**b**). Heatmap of significantly differently expressed miRNAs between study subgroups (**c**).

MiRNAs were filtered for those present in 100% of both groups, leaving 155 miRNAs for further analysis. PCA was performed to increase the general understanding of the whole miRNA signature (Figure 1a). Twenty-one miRNAs were found to demonstrate significant differences in expression between the refractory and the sensitive group (Table 2, Figure 1b). Overexpression of miR-215-5p, miR-143-3p, miR-19b-3p, miR-16-2-3p, miR-122-5p, miR-148a-3p, miR-22-5p, miR-30e-5p and miR-29b-3p was seen in the bortezomib-refractory patients compared to the bortezomib-sensitive patients (Table 2).

In contrast, reduced expression of miR-744-5p, miR-151a-3p, miR-376a-3p, miR-191-5p, miR-181a-5p, miR-766-3p, miR-30c-5p, miR-130a-3p, miR-409-3p, miR-1224-3p and miR-328-3p was observed among the bortezomib-refractory patients compared to the bortezomib-sensitive patients (Table 2 and Figure 1c). To select a panel of miRNAs discriminating between refractory and sensitive group, a multivariate logistic regression model was generated. Univariate logistic regression analysis found 10 miRNAs to be significantly associated with refractoriness (Table 3).

**Figure 2:**
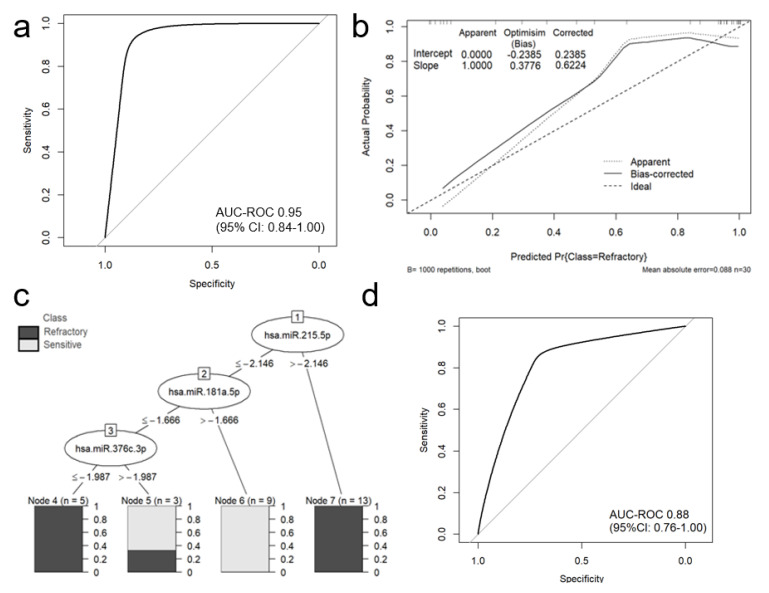
The performance of a classification miRNA-based model in predicting refractoriness to bortezomib-based treatment in MM patients. The area under the *ROC* curve for logistic regression model was estimated as 0.9474 (95% CI: 0.8421-1.000) (**a**). Calibration plot of the mi-RNA-based logistic regression model, including the apparent and bias-corrected measures by bootstrapping (**b**). In the calibration plot, the predicted probability of the model is represented on the *x*-axis and the observed proportion of refractoriness to bortezomib-based treatment is represented on the y axis. The 45° line indicates perfect congruity between the predicted probability and the observed proportion of bortezomib refractoriness. J48 decision tree with completely accurate (100%) model, separating refractory to bortezomib and sensitive MM patients (**c**). ROC analysis for 10-fold cross-validation (**d**).

A forward stepwise and backward stepwise selection approach were used to restrict the model to the most predictive miRNAs. Forward stepwise selection yielded a final model with the lowest AIC value. In the final multivariate logistic regression model, only miR-215-5p (odds ratio [OR] = 3.87; 95%CI 1.21–12.39; *p* = 0.02), miR-376c-3p (OR = 0.18; 95%CI 0.04–0.81; *p* = 0.03) and miR-181a-5p (OR = 0.07; 95%CI 0.01–0.97; *p* = 0.05) were associated with refractoriness to bortezomib treatment (Table 4). The combination of miR-215-5p, miR-376c-3p and miR-181a-5p could discriminate refractory from sensitive patients with a very high area under the ROC curve (AUC) of 0.95 (95% CI: 0.84–1.00); sensitivity, specificity and accuracy were estimated as 0.95, 0.91, and 0.93 (Figure 2a).

The Hosmer-Lemeshow test for goodness of fit indicated good fit of the model (*p* = 0.10). Internal validation of the selected panel model using bootstrap optimism corrected AUC suggests that the panel offers high discriminatory power when applied to independent samples (optimism-corrected AUC of 0.93, which was within the 95% CI for AUC on whole dataset).

We examined mi-RNAs expression between treatment-naive and previously treated patients- the results are given in Table S1. Briefly, among 21 differentially expressed miRNAs in bortezomib resistant-patients, only 2 miRNAs- hsa-miR-328-3p and hsa-miR-30e-5p were differentially expressed between treatment-naive and previously treated patients. These miRNAs were not statistically significant in univariate logistic regression models and were not included into building process of the final model.

The calibration plot of predicted and actual probability showed good correlation in low and in the intermediate probability area. The calibration plot, including the bias-corrected measures is given in Figure 2b. However, it appeared to have poor correlation in high probability areas. Additionally, a J48 (C4.5 classifier) decision tree was generated using miR-215-5p, miR-376c-3p and miR-181a-5p; this tree accurately separated refractory and sensitive patients in a training set (Figure 2c). ROC analysis for 10-fold cross-validation demonstrated preserved good discriminative ability of the model with AUC 0.88 (95%CI: 0.76–1.00) (Figure 2d).

**Figure 3:**
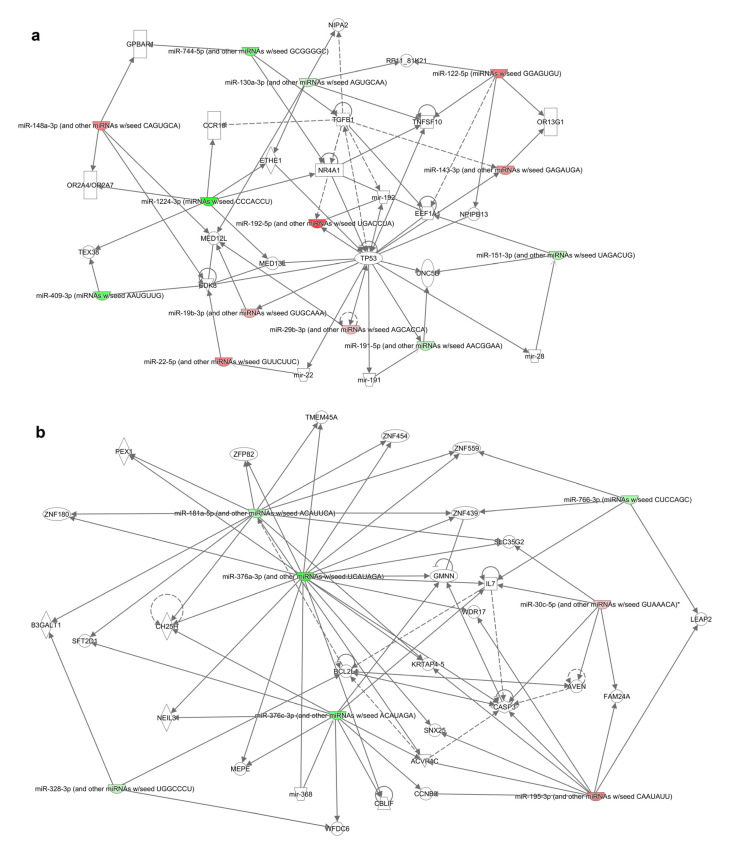
Gene networks (**a**,**b**) identified from the differentially expressed miRNA’s target genes list generated by IPA. Solid and dotted lines indicate direct and indirect relationships, respectively. Color intensity indicates upregulation (red) and downregulation (green).

Moreover, the resultant differentially expressed miRNAs were analyzed through the use of Ingenuity Pathway Analysis. MiRNA targets were found using IPA’s miRNA target filter for experimentally validated (Table S2) and putative predicted targets (high confidence level- Table S3)—*in silico* analyses. We have also generated gene networks based on miRNAs exhibiting significant changes in patients refractory to bortezomib-based treatment (Figure 3).

## 3. Discussion

Serum miRNAs, with their noticeable stability and unique expression pattern are robust novel non-invasive biomarkers that can be used for cancer detection and identifying disease characteristics [25]. To our knowledge, this is the first study to demonstrate that the serum expression of several miRNAs differs between patients refractory to bortezomib and those who are sensitive. The present study identifies 21 serum miRNAs differentially expressed in patients with MM according to the bortezomib sensitivity based on univariate analysis. The serum levels of nine miRNAs were lowered, and another 12 were elevated, in bortezomib-refractory patients compared to the bortezomib-sensitive patients. Few studies have demonstrated the significance of miRNAs in MM patients, especially in the context of drug resistance. However, some of them have been previously reported as important in the biology and clinical implications of MM [18,26,27,28,29,30,31].

In the present study, higher serum levels of miR-215-5p, miR-143-3p, miR-19b-3p, miR-16-2-3p, miR-122-5p, miR-148a-3p, miR-22-5p, miR-30e-5p and miR-29b-3p were seen in bortezomib-refractory patients compared to bortezomib-sensitive patients (Table 2). MiR-215-5p, miR-30e-5p and miR-29b-3p have been found to play important roles in the biology of MM. In a previous study, miR-215-5p was negatively correlated with runt-related transcription factor 1 (RUNX1) expression in MM clinical bone marrow samples [32]. In addition, miR-215-5p overexpression increased cell proliferation and inhibited MM cell apoptosis by targeting RUNX1 and suppressing the activation of the PI3K/AKT/mTOR pathway. These findings indicate that overexpression of miR-215-5p inhibits the proliferation of MM cells and promotes their apoptosis, while also inducing the cell cycle progression from G1/S phase of MM cells; hence, miR-215-5p appears to be an anti-oncogene of MM. MiR-30e-5p was differentially expressed in MM patients with and without extramedullary relapse [33].

In another study in which fluorescence in situ hybridization was used, miR-30e-5p was found to be a biomarker for distinguishing MM with 1q21 gains from MM [33]. In vitro and in vivo studies both suggest that miR-29b plays a tumor suppressor function. MiR-29b-3p is involved in bortezomib resistance in MM patients. Pan et al. reported recently that LncRNA H19 induces bortezomib resistance by targeting MCL-1 via miR-29b-3p [26]. It is suggested, that H19/miR-29b-3p/MCL-1 may be a novel and promising therapeutic target for coping with drug resistance in MM treatment [34,35]. An earlier study performed in MM showed that miR-143-3p expression is regulated by long non-coding RNA Sox2 overlapping transcript (SOX2OT) [35].

To the best of our knowledge, six of the miRNAs overexpressed in bortezomib refractory patients (miR -143-3p, miR-19b-3p, miR-16-2-3p, miR-122-5p, miR-148a-3p and miR-22-5p) have not been investigated in MM patients yet. However, the results of several studies indicate the involvement of these molecules in the biology of other neoplasms. MiR-143-3p was identified as the key specific mediator of KIAA1429 expression in osteosarcoma cells [36]. In addition, it is also involved in the regulation of cell proliferation in cervical cancer, esophageal cancer, breast cancer, lung cancer and other neoplasms [37,38,39,40]. MiR19b-3p has also been implicated in the pathogenesis of several cancers in humans [41,42]. Jiang et al. found miR-19b-3p to promote cell proliferation and induce resistance to oxaliplatin-based chemotherapy in colon cancer in vitro, and promote tumorigenesis in vivo [43]; these findings are similar to our present observations regarding higher expression of this miRNA in bortezomib-refractory MM patients. MiR-19b-3p is highly expressed in colon cancer tissues, and although its downregulation has been associated with decreased cell proliferation, it has not been found to have any effect on tumor invasion [43]. Elsewhere, overexpression of miR-19b-3p was observed to decrease the sensitivity of nasopharyngeal carcinoma cells to radiotherapy, whereas down-regulation resulted in the opposite results [44]. In addition, in patients with colon cancer miR-19b-3p is an independent prognostic factor associated with disease-free survival and overall survival. These results suggest a role of miR-19b-3p as potential therapeutic target against chemotherapy resistance, including MM.

MiR-16-2-3p demonstrates strong tumor suppressive and antimetastatic properties in osteosarcoma and other neoplasms [45]. Several studies suggest that miR-122-5p may be a potential prognostic factor and therapeutic target for patients with pancreatic ductal adenocarcinoma, hepatocellular carcinoma and other neoplasms [46,47]. In addition miR-122-5p acts as a tumor suppressor in gastric cancer progression and inhibits the proliferation, migration, and invasion of gastric cancer cells [48,49]. Biologically, miR-148a-3p influences cellular differentiation and the cellular development of several tumors; it also promotes the differentiation of activated B cells to plasma cells and increases survival of plasma cells by inhibiting the transcription factors BACH2 and MITF [50]. MiR-148a-3p was upregulated in some cancers and downregulated in others [51]. In particular, increased miR-148a-3p serum level is observed in prostate cancer, glioma and osteosarcoma [52]. Upregulation of miR-22-5p was has also been recorded in cholangiocarcinoma cell lines [53]. In addition, miR-22 acts as a novel biomarker for non-small cell lung cancer and inhibits tumor growth and metastasis [54,55]. miR-22 has also been found to be significantly associated with osteosarcoma grade, impaired osteosarcoma cell proliferation and epithelial-mesenchymal transition progression [56].

The serum levels of miR-744-5p, miR-151a-3p, miR-376a-3p, miR-191-5p, miR-181a-5p, miR-766-3p, miR-30c-5p, miR-130a-3p, miR-409-3p, miR-1224-3p and miR-328-3p, which were lower in the refractory group than the sensitive patients (Table 2). MiR-181a-5p is a direct target of colon cancer associated transcript-1 (CCAT1), and its repression could rescue the inhibition of CCAT1 knockdown on MM progression [57]. MiR-181a-5p has previously been found to be a target of LncRNA MALAT1, whose interference decreased the expression of miR-181a-5p and inhibited the proliferation and adhesion of myeloma cells [58].

Although no previous studies have confirmed that other miRNAs are down-regulated in bortezomib refractory MM, they are known to be involved in the development of various cancers, and some of them play an important role in cancer progression. Overexpression of miR-744-5p was found to inhibit the proliferation, migration, and invasion of cancer cells in epithelial ovarian cancer, non-small cell lung cancer and in nasopharyngeal carcinoma [59,60]. MiR-376a is silenced in several types of cancer, suggesting its role as tumor suppressor for the development and progression of cancer [61,62]. MiR-191-5p is believed to act as a tumor promoter: elevated miR-191-5p levels have been correlated with the advanced stages, metastasis, and high pathological grade of hepatocellular carcinoma [63]. Serum exosomal miR-766-3p is also thought to be a prognostic marker for esophageal squamous cell carcinoma with its serum expression being associated with poor prognosis and a significantly worse survival [64]. In addition, miR-766 acts as an oncogene in the development of colorectal cancer and its overexpression promotes cell proliferation in this disease [65]. In addition, higher expression of miR-766-3p was found in the plasma of patients with colorectal cancer compared to healthy controls [66]. MiR-30c-5p is involved in many biological events, including cell apoptosis, growth, and differentiation. In a recent study, decreased expression of miR-30c-5p was associated with an unfavorable course of the renal cell carcinoma [67]. MiR-130a-3p, a member of the miR-130 family, is an important regulator of tumor progression and metastasis [68]. While the members of the miR-130 family are upregulated in several cancers and promote cell proliferation, they have also been found to be downregulated and inhibit cell proliferation in a number of studies [25,69,70,71,72,73].

Some of the miRNAs identified in our study as being differentially expressed in bortezomib refractory and sensitive MM patients are known to be potentially important for prediction of response to treatment in other cancers. MiR-151a-3p could predict the response to azacitidine treatment in myelodysplastic syndromes when tested in combination with miR-423-5p, miR-126-3p miR-125a-5p, and miR-199a-3p [74]. Using Cox regression and risk-score analysis, Ji et al. identified miR-328-3p and three additional miRNA signature (miR-652-3p, miR-342-3p and miR-501-3p) that can be used for the prediction of treatment outcome including tumor relapse and the OS of patients with colorectal cancer [75]. In the present study, these miRNAs were found to be less useful for the prediction of bortezomib refractoriness in MM patients; however, the final version of the multivariate logistic regression model constructed to predict refractoriness to bortezomib-based treatment in MM patients found only miR-215-5p, miR-376c-3p and miR-181a-5p to be associated with refractoriness to bortezomib treatment (Table 4, Figure 2a). Our findings, indicating the differential expression of miRNAs between bortezomib-refractory and bortezomib-sensitive patients, suggest that these circulating miRNAs may play an important role in the correct treatment selection in MM patients.

Finally, combining miRNAs with anti-MM drugs may improve treatment efficacy and prevent drug resistance among patients with MM [18,76,77,78]. Theoretically, it is possible that inhibition of miRNAs overexpressed in bortezomib-refractory MM can improve drug sensitivity. The possible synergistic effect between miRNA inhibition and anti-MM drugs has been investigated in preclinical studies. Inhibition of miR-221/222 reduced the drug resistance of MM1R MM cells to dexamethasone via upregulation of the pro-apoptotic PUMA in vitro and improved mouse survival in vivo [77].

Elsewhere, miR-221/222 overcame melphalan resistance and triggered apoptosis of MM cells in vitro: Gulla et al. demonstrated that inhibition of miR-221/222 via LNA-i-miR-221 triggers cell apoptosis in vitro and restores drug sensitivity in melphalan-refractory MM cells [22]. In vivo treatment of SCID/NOD mice bearing human melphalan-refractory MM xenografts with systemic locked nucleic acid (LNA) inhibitors of miR-221 (LNA-i-miR-221) combined with melphalan increased the antitumor effects seen in tumors retrieved from treated mice [22]. In another study, miR-29b replacement disrupted the autophagy pathway and synergistically enhanced the antimyeloma effect of bortezomib in bortezomib-resistant MM cells, increasing the antimyeloma effect of bortezomib [79]. In addition, MiR-21 inhibition combined with the cytotoxic drugs dexamethasone, doxorubicin, or bortezomib inhibited myeloma cell survival more effectively than either treatment alone [80]. These results suggest that influencing miRNA expression may be an effective strategy for improving anti-myeloma therapy.

Our findings demonstrated that serum expression of several miRNAs differs between patients who are refractory to bortezomib and those who are sensitive. However, it is important to examine the roles of these miRNAs in the regulation of recently-identified genes associated with bortezomib resistance, such as *PSMB5*, *CDK5* and *CYP1A1*, in MM cells [81,82,83]. It has been reported previously that miRNAs can modulate the expression of genes responsible for bortezomib refractoriness in MM cells; for example, CDK5 expression may be related to miR-27a-5p activity [84]. However, to determine the degree of this modulation, further studies are needed of the miRNAs identified as being associated with bortezomib refractoriness.

## 4. Materials and Methods

### 4.1. Patients

A total of 30 MM patients who were admitted to the Department of Hematology, Copernicus Memorial Hospital, Lodz, Poland were included in the study. The group of patients included 18 men and 12 women with a mean age of 64 years (range, 39–81 years). Their demographic, clinical and laboratory details are shown in Table 1. All of the patients received bortezomib treatment as the first line treatment or in progression after previous therapy. No patients had received bortezomib-based therapy prior to the study. The participants were classified according to their response to bortezomib-based therapy as either bortezomib sensitive or bortezomib refractory [5].

Response to treatment and relapse/progression events were classified according to the International Myeloma Working Group (IMWG) [85,86]. Sensitive patients had CR or PR after bortezomib-based therapies longer than six months after treatment discontinuation [86,87,88].

The study was conducted according to good clinical and laboratory practice rules and the principles of the Declaration of Helsinki. All procedures were approved by the local ethical committee (The Ethical Committee of the Medical University of Lodz, No RNN/103/16/KE). Each patient signed the informed consent for all examinations and procedures.

### 4.2. Serum Collection

Venous blood samples were collected from MM patients before treatment with bortezomib based regimens. Peripheral blood was collected in serum separating tubes and processed within 2 h of collection by centrifugation at 2400× *g* for 10 min. Serum samples were stored at −80 °C. for further use.

### 4.3. miRNA Isolation

Isolation of total RNA including miRNA was performed from serum using the miRNeasy Serum/Plasma Advanced Kit (Qiagen, Germantown, MD, USA) according to the manufacturer’s protocol. Samples of 2 μL of the final eluate was used as the sample input. To allow for normalization of sample-to-sample variation in RNA isolation, prior to purification, each serum sample was spiked with UniSp2 (2 fmol), UniSp4 (0.02 fmol), UniSp5 (0.00002 fmol), each at a different concentration in 100-fold increments and UniSp6 (0.075 fmol) and cel-miR-39-3p (0.001 fmol) as a positive control for cDNA synthesis. RNA quality was determined with Agilent High Sensitivity RNA ScreenTape using 2200 TapeStation (Agilent Technologies, Santa Clara, CA, USA). Directly after isolation RNA was subjected to the reverse transcription process.

### 4.4. Determination of MicroRNA Profile

MmiRNA profiling was performed by miRCURY LNA RT Kit (Qiagen) and used to prepare the reverse transcription reaction according to the manufacturer’s recommendations. Real-time PCR was performed on a LightCycler480 Real Time PCR System (Roche, Pleasanton, CA, USA) using and miRCURY LNA miRNA miRNome PCR Panel I and II. This array enables accurate quantitation of 752 human microRNAs.

### 4.5. Reverse Transcriptase Reaction

Mature miRNAs were polyadenylated by poly(A) polymerase and reverse transcribed into cDNA using oligo-dT primers. Polyadenylation and reverse transcription were performed in parallel in the same tube. The oligo-dT primers have a 3′ degenerate anchor that allows amplification of mature miRNA in the real-time PCR step. MiRCURY LNA Reverse Transcription Kit was purchased from Qiagen and used to synthesize cDNA according to the guidelines provided by the manufacturer. Two µL of isolate was added to the reaction tube to make up a final volume of 10 μL reaction mix. The reaction took place at 42 °C for 60 min and were then inactivated at 95 °C for 5 min in thermal cycler.

### 4.6. Real Time PCR (qPCR)

The expression of 752 miRNAs were determined in all patients using miRCURY LNA miRNome Human PCR Panel I and II (Qiagen). Real-time PCR was performed on LightCycler480 II (Roche) instrument. Three µL of cDNA template (diluted 1:60) was used in each PCR reaction. The reaction was performed at 95 °C for 2 min, followed by 45 amplification cycles at 95 °C for 10 s and 56 °C for 1 min. Fluorescence was measured after each cycle. Relative quantification of mRNA was determined by a comparative Ct method.

### 4.7. Statistical Analysis

#### 4.7.1. Data Preparation

miRNAs were filtered for miRNAs present in 100% of both groups, leaving 155 miRNAs to further analysis. After *miRNA* selection, batch correction was performed using ComBat [89]. The stability of miRNAs was measured by NormiRazor [90,91]. This is an integrative tool which implements existing normalization algorithms (geNorm, NormFinder and BestKeeper) in a parallel manner. The data was normalized by using used the mean expression value of three miRNAs in a given sample (miR-23b-3p, miR-151a-5p and miR-152-3p), which proved to be the most stable normalization factor (according to NormiRazor). The formula used to calculate the normalized Ct values was:

Normalized ΔCt = mean Ct of miR-23b-3p, miR-151a-5p and miR-152-3p—Ct miRNA.

This approach results in higher values for higher miRNA expression enabling easy biomarker interpretation. Normalized ΔCt for all samples and with class assignments were provided as Table S4.

#### 4.7.2. Analysis

Nominal variables were expressed as percentages and analyzed using the Chi-square test with appropriate corrections if needed: the Yates correction for continuity or Fisher’s exact test. The normality of the distribution of continuous variables was verified with the Shapiro-Wilk test. For continuous variables, the difference between two groups was evaluated using a two-sided independent Student’s t test when the data was normally distributed, and with the Mann-Whitney U test when the assumption of normality was not met or if the variable was ordinal. Continuous variables were presented as mean ± standard deviation (SD) or medians with 25% to 75% values according to the data distribution. For visual representation of multidimensional data, principal component analysis (PCA) and unsupervised hierarchical clustering were performed.

For a more comprehensive analysis, a logistic regression model was generated that used the refractoriness to a bortezomib-based regimen as outcome and miRNAs as predictors. The variables were preselected for the development of the classification model using Student’s t-test. All miRNA with FDR < 0.2 were chosen as candidate variables in this step. We estimated both a univariate model for each of the selected miRNA and a multivariate model that included all selected predictors. Forward stepwise and backward stepwise selection approaches were used to restrict the model to the most predictive miRNAs. The model with the lowest AIC (The Akaike information criterion) value was chosen as the final model. The predictive power of the final model was evaluated by receiver operating characteristics (ROC) and area under the curve (AUC) analysis to determine the ability of the biomarker panel to accurately predict refractoriness to the bortezomib-based treatment regimen. The goodness of fit of the model was tested with the Hosmer-Lemeshow statistic, where high p values indicate a good fit.

#### 4.7.3. Internal Validation

Bootstrap samples were used to test for possible overfitting by determining optimism values on calibration measures. The bootstrap analysis was performed on 1000 different samples of the same sample size drawn with replacements from the original sample. Optimism, which is a measurement of internal model validation that refers to the absolute magnitude of bias, equals the difference between respective statistics of the bootstrap sample (bootstrap performance) and the bootstrap model in the original sample (test performance) [92]. Bias-corrected (bc) AUC was calculated as the mean of AUC metrics derived from 1000 bootstrap samples. Additionally, J48 (C4.5 classifier) decision tree was generated and tested in 10 times repeated 10-fold cross-validation manner to reduce the bias and variance of the estimations. Lastly, to find further biological insights, the resultant differentially expressed miRNAs were analyzed through the use of IPA (Ingenuity Pathway Analysis, Qiagen Inc.).

All statistical analyses were conducted using Statistica Version 13.1 (TIBCO, Palo Alto, CA, USA) and R programming language (version 4.0.2). Most of the analysis utilized our *miRNAselector* R package [93] P values lower than 0.05 were considered statistically significant. Benjamini and Hochberg multiple comparisons correction was used to adjust individual raw *p* values.

## 5. Conclusions

In conclusion, our data demonstrate differential serum miRNA expression between patients with MM who are sensitive to bortezomib-based therapies and those who are not. To our knowledge, this is the first study to demonstrate such differences between these two groups of patients. These results indicate a promising therapeutic target for coping with drug resistance in MM treatment. In addition, we describe the development of a multivariate regression model for predicting refractoriness to bortezomib-based treatment in MM patients. Our findings provide evidence that combined expression levels of miR-215-5p, miR-376c-3p and miR-181a-5p can be used to predict the response to bortezomib treatment. Internal validation of the selected panel suggests that the discriminatory power of the panel will be high when applied to independent samples. In addition, miRNAs are promising therapeutic target for coping with drug resistance in MM treatment. However, the clinical and biological importance of these findings need further investigation.

## Figures and Tables

**Table 1:** The characteristics of the MM patients treated with Bortezomib-based therapy.

Variable	Total	Refractory	Sensitive	*p*-Value
Number of patients	30	19	11	-
Gender M F	18 (60.0) 12 (40.0)	12 (63.2) 7 (36.8)	6 (54.5) 5 (45.5)	0.73
Age at diagnosis Mean ± SD (range)	64.0 ± 9.9 (39–81)	65.2 ± 10.6 (39–81)	62.2 ± 8.6 (46–76)	0.44
Bortezomib-based regimen VCD VMP VTD	27 (90.0) 2 (6.7) 1 (3.3)	16 (84.2) 2 (10.5) 1 (5.3)	11 (100.0) 0 0	0.36
Paraprotein IgG kappa IgG lambda LCD lambda IgA kappa LCD kappa IgA lambda	11 (36.7) 7 (23.3) 6 (20.0) 4 (13.3) 1 (3.3) 1 (3.3)	7 (36.8) 4 (21.1) 3 (15.8) 4 (21.1) 0 1 (5.3)	4 (36.4) 3 (27.3) 3 (27.3) 0 1 (9.1) 0	0.36
Bone involvement at diagnosis	14 (46.7)	9 (47.4)	5 (45.5)	1.00
Calcium > 2.75 mmol/L at diagnosis	1 (3.3)	1 (5.3)	0	1.00
Calcium > 2.52 mmol/L at diagnosis	4 (13.3)	2 (10.5)	2 (18.2)	0.94
HB < 10 g/dL at diagnosis	12 (40.0)	7 (36.8)	5 (45.5)	0.87
Creatinine > 2 mg/dL at diagnosis	5 (16.7)	2 (10.5)	3 (27.3)	0.59
Previous treatment	6	6	0	0.06
ISS	I- 9 (30.0) II- 6 (20.0) III- 15 (50.0)	I- 6 (31.6) II- 4 (21.1) III- 9 (47.4)	I- 3 (27.3) II- 2 (18.2) III- 6 (54.5)	0.96
CRP > 5 mg/L	7 (23.3)	4 (21.1)	3 (27.3)	0.72
Beta−2 microglobulin (>3 mg/L)	20 (66.7)	12 (63.2)	8 (72.7)	0.61
LDH > 240 U/L	4 (13.3)	3 (15.8)	1 (9.1)	0.81
Cytogenetics amp(1q) *IGH rearrangements* del(17p) del(13q) t(4;14) t(11;14) del(1p) t(14;16) t(14;20)	N = 13 8 (61.5) 6 (46.2) 4 (30.8) 4 (30.8) 3 (23.1) 1 (7.7) 1 (7.7) 0 0	N = 8 5 (62.5) 5 (62.5) 2 (25.0) 1 (12.5) 3 (37.5) 1 (12.5) 0 0 0	N = 5 3 (60.0) 1 (20.0) 2 (40.0) 3 (60.0) 0 0 1 (20.0) 0 0	1.00 0.24 1.00 0.14 0.23 1.00 0.42

Abbreviations: LCD—light chain disease; VCD— bortezomib, cyclophosphamide and dexamethasone; VMP—bortezomib, melphalan and prednisone; VTD—bortezomib, thalidomide, dexamethasone; ISS—International Staging System.

**Table 2:** List of miRNAs with significantly different expression between groups in univariate analysis.

miRNA	Average Expression ± SD - Refractory Group	Average Expression ± SD - Sensitive Group	Fold Change	log2FC	*p*-Value	FDR
hsa-miR-744-5p	−5.03 ± 0.80	−3.73 ± 0.87	0.41	−1.30	0.0006	0.0823
hsa-miR-215-5p	−1.82 ± 1.29	−3.58 ± 1.20	3.38	1.75	0.0011	0.0823
hsa-miR-151a-3p	−1.72 ± 0.63	−0.94 ± 0.61	0.58	−0.77	0.0031	0.1163
hsa-miR-376a-3p	−3.28 ± 2.02	−1.44 ± 1.08	0.28	−1.83	0.0031	0.1163
hsa-miR-143-3p	−0.65 ± 0.86	−1.66 ± 0.85	2.02	1.01	0.0051	0.1163
hsa-miR-19b-3p	3.91 ± 0.76	3.16 ± 0.59	1.69	0.75	0.0056	0.1163
hsa-miR-16-2-3p	−1.43 ± 0.95	−2.65 ± 1.08	2.33	1.22	0.0057	0.1163
hsa-miR-191-5p	0.40 ± 0.66	1.09 ± 0.56	0.62	−0.68	0.0061	0.1163
hsa-miR-181a-5p	−1.97 ± 0.75	−1.15 ± 0.75	0.57	−0.82	0.0088	0.1492
hsa-miR-376c-3p	−3.12 ± 1.26	−1.81 ± 1.21	0.40	−1.31	0.0104	0.1579
hsa-miR-122-5p	1.35 ± 1.80	0.12 ± 0.72	2.35	1.23	0.0137	0.1893
hsa-miR-766-3p	−3.53 ± 1.09	−2.76 ± 0.62	0.59	−0.77	0.0199	0.2497
hsa-miR-30c-5p	−0.89 ± 0.65	−0.40 ± 0.44	0.71	−0.49	0.0214	0.2497
hsa-miR-148a-3p	1.70 ± 1.60	0.57 ± 0.99	2.20	1.13	0.0235	0.2555
hsa-miR-22-5p	−3.27 ± 0.99	−4.46 ± 1.41	2.28	1.19	0.0255	0.2584
hsa-miR-130a-3p	−0.19 ± 0.63	0.36 ± 0.62	0.68	−0.55	0.0298	0.2830
hsa-miR-409-3p	−5.59 ± 2.21	−4.18 ± 1.31	0.38	−1.41	0.0361	0.3230
hsa-miR-30e-5p	0.78 ± 0.67	0.21 ± 0.70	1.49	0.57	0.0396	0.3230
hsa-miR-1224-3p	−6.62 ± 2.04	−4.67 ± 2.51	0.26	−1.95	0.0418	0.3230
hsa-miR-328-3p	−3.26 ± 0.45	−2.61 ± 0.89	0.64	−0.65	0.0425	0.3230
hsa-miR-29b-3p	−2.83 ± 0.78	−3.46 ± 0.80	1.55	0.63	0.0483	0.3494

**Table 3:** Univariate logistic regression analysis for predicting refractoriness to bortezomib-based treatment in MM patients.

miRNA	Coefficient	OR	95% CI		*p*-Value
			Lower	Upper	
hsa-miR-215-5p	1.13	3.08	1.28	7.41	0.0120
hsa-miR-744-5p	−2.81	0.06	0.01	0.57	0.0146
hsa-miR-143-3p	1.42	4.14	1.31	13.09	0.0157
hsa-miR-151a-3p	−2.56	0.08	0.01	0.64	0.0172
hsa-miR-16-2-3p	1.30	3.68	1.26	10.76	0.0174
hsa-miR-376a-3p	−0.84	0.43	0.21	0.87	0.0194
hsa-miR-19b-3p	1.57	4.78	1.27	17.98	0.0205
hsa-miR-376c-3p	−0.83	0.44	0.21	0.88	0.0210
hsa-miR-181a-5p	−1.51	0.22	0.06	0.80	0.0219
hsa-miR-191-5p	−2.61	0.07	0.01	0.79	0.0311
hsa-miR-122-5p	0.78	2.18	0.98	4.86	0.0557

**Table 4:** Final multivariate regression model for predicting refractoriness to bortezomib-based treatment in MM patients.

miRNA	Coefficient	OR	95% CI	*p* Value
hsa-miR-215-5p	1.35	3.873	1.210–12.389	0.0225
hsa-miR-376c-3p	−1.72	0.178	0.039–0.806	0.0251
hsa-miR-181a-5p	−2.67	0.069	0.005–0.973	0.0477

## Supplementary Materials

The following are available online at https://www.mdpi.com/2072-6694/12/9/2569/s1, Table S1: differentially expressed mi-RNAs between treatment-naive and previously treated patients- among 21 differentially expressed miRNAs in bortezomib resistant-patients, only 2 miRNAs- hsa-miR-328-3p and hsa-miR-30e-5p were differentially expressed between treatment-naive and previously treated patients, Table S2: Putative miRNA targets found using Ingenuity miRNA target filter for experimentally validated targets, Table S3: Putative miRNA targets found using Ingenuity miRNA target filter for experimentally validated targets and putative predicted targets (high confidence level), Table S4: Normalized ΔCt for all samples and with class assignments.

## Abbreviations

BM	bone marrow
Bcl-2	B-Cell Leukemia/Lymphoma 2
FISH	fluorescent in situ hybridization
MM	Multiple myeloma
MiRNAs	MicroRNAs
MeV	Multi Experiment Viewer
Mcl-1	myeloid cell leukemia sequence 1
MGUS	monoclonal gammopathy of unknown significance
qRT-PCR	quantitative real-time polymerase chain reaction
CLL	lymphocytic leukemia
CR	complete response
VGPR	very good partial response
OS	overall survival
PCR	*polymerase chain reaction*
PFS	progression free survival
PR	partial response
ROC	receiver operating characteristics
RUNX1	runt-related transcription factor 1
AUC	area under the curve
